# Combining biomarkers to predict the disease activity of graves’ ophthalmopathy: a combinatory model of the NLR, TRAb and FT4

**DOI:** 10.3389/fendo.2025.1546211

**Published:** 2025-06-20

**Authors:** Tongxin Niu, Lujue Wang, Jing Deng, Yuxian Shi, Yating Liu, Boding Tong, Xin Qi, Dan Cao, Yunping Li

**Affiliations:** ^1^ Department of Ophthalmology, The Second Xiangya Hospital of Central South University, Changsha, Hunan, China; ^2^ Hunan Clinical Research Center of Ophthalmic Disease, Changsha, Hunan, China; ^3^ National Clinical Research Center for Metabolic Diseases, The Second Xiangya Hospital of Central South University, Changsha, Hunan, China

**Keywords:** NLR-neutrophil-to-lymphocyte Ratio, TRAb-thyrotrophin receptor antibody, FT4-free tetraiodothyronine, biomarkers, disease activity, graves' ophthalmopathy-GO

## Abstract

**Purpose:**

The aim of this study was to identify potential biomarkers associated with active Graves’ ophthalmopathy (GO) and develop a model for predicting the occurrence and progression of active GO.

**Methods:**

A retrospective study was conducted on 220 GO patients (n=120 in the active phase, n=100 in the stable phase) and 70 healthy controls. Laboratory and other clinical indicators were compared in GO patients at different stages and healthy controls. A multivariate regression analysis model was used to analyze the clinical risk factors affecting the occurrence and progression of active GO, and a predictive model based on risk factors was established.

**Results:**

Higher WBC, NEU, NLR, TRAb and FT4 levels (all p<0.005) were detected in patients with active GO than those in inactive GO. The CAS score of the GO patients was positively correlated with the WBC (r = 0.155, p = 0.003), NEU (r = 0.165, p = 0.002), NLR (r = 0.134, p = 0.010), FT3 (r = 0.117, p = 0.031), FT4 (r = 0.139, p = 0.011), and TRAb (r = 0.160, p = 0.004) counts and negatively correlated with the TSH level (r = - 0.114, p = 0.043). Multivariate regression analysis revealed that the NLR, TRAb, and FT4 were significant risk factors for GO progression (all p < 0.05). The cut-off levels for predicting active GO were 8.71 IU/L for TRAb (AUC=0.643, sensitivity 0.58, specificity 0.74), 19.82 ng/dL for FT4 (AUC=0.606, sensitivity 0.41, specificity 0.75), and 2.405 for the NLR (AUC=0.597, sensitivity 0.50, specificity 0.75). A predictive model including these risk factors was built (the cutoff value was 0.6, the AUC was 0.716, the sensitivity was 0.533, and the specificity was 0.808).

**Conclusion:**

The laboratory biomarkers for the occurrence and progression of active GO include the NLR, FT4, and TRAb. We have developed a predictive combined model that may assist in timely assessment of GO activity and progression, and provide clues for future development of facile tools to predict GO activity.

## 1 Introduction

Graves’ ophthalmopathy, the most common extrathyroidal manifestation of Graves’ disease (GD), features inflammatory changes and volume expansion of orbital tissues causing proptosis, eyelid retraction, edema, restricted eye movement, and diplopia. Severe cases may develop vision-threatening complications including corneal exposure and optic neuropathy (1–3). The active phase has a decisive impact on the disease outcome (4). If the activity of GO is not timely identified and treated, severe retrobulbar tissue edema may compress the optic nerve, affecting vision and potentially leading to irreversible vision loss (5). Furthermore, persistent exophthalmos and incomplete eyelid closure may result in exposure keratitis, ultimately causing permanent vision damage. Patients often experience a significant decline in appearance and quality of life due to symptoms such as eye swelling, protrusion, and pain, which in turn affect their social interactions, work, and even daily activities. Thus, proper evaluation of disease activity and severity is crucial for timely diagnosis, risk stratification, treatment planning, and outcome prediction.

In clinical practice, clinicians rely mainly on the subjective clinical activity score (CAS) system to quantify the severity and activity of the disease. Disease activity was assessed via the CAS of 7 items: spontaneous retrobulbar pain, gaze-evoked pain, eyelid erythema, conjunctival hyperemia, eyelid swelling, chemosis and inflammation of the caruncle/plica. One point was given for each sign, and CAS was defined as ≥3/7 for the active-phase group and <3 for the inactive-phase group. However, the CAS system has been criticized for being too binary, being subjective, and lacking in sensitivity, which can easily delay the diagnosis and treatment of GO in a clinical setting and is not adequate for monitoring treatment response. Although more complex scoring systems have been introduced, such as VISA [V (vision, DON); I (inflammation, congestion); S (strabismus, motility restriction); A (appearance, exposure)], modified NOSPECS [N (no signs or symptoms); O (only signs, symptoms); S (soft tissue involvement); P (proptosis); E (extraocular muscle involvement); C (corneal involvement); S (sight loss)] and EUGOGO scoring tools, fundamentally, they remain subjective and are cumbersome to administer (6, 7), which leads to misjudgment of the activity of GO. Incorrect judgment can result in delayed treatment for patients, aggravating their condition and causing a heavy blow to their physical and mental health. Therefore, it is necessary to look for a cost-effective, easily accessible and commonly used index, such as laboratory biomarkers.

A few studies have shown that some biomarkers, including white blood cell (WBC) count, neutrophil count (NEU), thyroid-stimulating hormone receptor antibodies (TRAb) (8, 9) and low-density lipoprotein (LDL) (10), are closely related to GO activity. Recently, a high neutrophil-to-lymphocyte ratio (NLR) (11, 12) has also been identified as a potential marker involved in the development of active GO. These findings suggest that the NLR can be used as a prognostic marker for relapse in patients with GD after antithyroid drug (ATD) therapy. A high NLR is associated with relapse in Graves’ disease patients after antithyroid drug therapy with active GO (13). However, previous studies have primarily focused on exploring the expression differences of individual factors in different stages of GO, with few attempts to integrate multiple relevant factors to improve the prediction of disease activity and progression. Moreover, there is a notable lack of simple and practical clinical prediction models. This single-factor research paradigm significantly limits its clinical translational value and fails to attract sufficient attention from clinicians, rendering the clinical significance of existing findings rather limited. Therefore, it is particularly urgent to establish a novel, efficient, and clinically applicable predictive model for GO activity.

In this study, we retrospectively identified laboratory biomarkers associated with disease activity and severity in patients with GO and established a model based on these biomarkers to predict GO disease activity and severity. This model may provide a new tool that can be easily applied in clinical practice to predict the occurrence and progression of active GO and hopefully provide timely recommendations for the precise treatment of GO. These findings lay the foundation for the upcoming prospective study of the application of laboratory biomarkers to predict the activity of GO patients, and may provide a new reference for clinicians to quickly and accurately determine GO activity in the future.

## 2 Methods

### 2.1 Statement of ethics

This study was designed as a retrospective, observational study and was approved by the ethical institutional review board of the second Xiangya Hospital of Central South University, China (LYEC2025-K0042). Owing to the retrospective nature of the study, informed consent was not needed, and patients’ data were used anonymously. The research was conducted in accordance with Good Clinical Practice (Declaration of Helsinki of 1975).

### 2.2 Participant selection

Patients who met the criteria for GO were enrolled between January 2010 and December 2023 at the second Xiangya Hospital of Central South University. The inclusion criteria in the study were as follows: (a) typical ocular symptoms (e.g., eyelid retraction, exophthalmos, strabismus, diplopia); (b) abnormal thyroid function or thyroid-related antibodies; and (C) imaging findings (e.g., extraocular muscle thickening). The exclusion criteria were as follows (1): age less than 18 years (2); clinical evidence of infection; (3) hematological system diseases; (4) history of ocular surgery or trauma; (5) use of ocular or systemic medications that have been proven to affect blood counts [Specified exact drug classes and washout periods: (a) corticosteroids (≥1 month prior), (b) immunosuppressants (≥3 months), (c) chemotherapy (≥6 months), (d) granulocyte-stimulating factors (any history)]; (6) use of glucocorticoids in the past 6 months; (7) pregnancy; (8) history of malignant tumors; and (9) history of thyroidectomy and radioactive iodine therapy. Patients with incomplete medical records were also excluded from the study. The healthy control group were required to: (1) demonstrate euthyroidism (TSH 0.4-4.0 mIU/L, normal FT4), (2) have no personal/family history (1st-degree) of thyroid autoimmunity, (3)have no smoking history, (4) show normal inflammatory markers (CRP<3 mg/L).

Finally, 220 patients with GO and 70 healthy individuals were included in the analysis. Medical history and demographic features were recorded for all patients and healthy controls. For GO patients, the results from ophthalmological and orbital examinations were retrospectively obtained from patient files.

For the validation of the prediction model, a retrospective analysis was conducted on 15 patients who responded well to intravenous methylprednisolone (IVMP) (using the same inclusion and exclusion criteria as before) between January and August 2024. The IVMP dosing scheme was 500 mg for three consecutive days (based on EUGOGO guidelines and tailored in selected cases depending on comorbidities and side effects). A beneficial response to IVMP treatment was defined as (1) achieving a total CAS of < 3 in both eyes or (2) an improvement of ≥ 2 points in one eye without concomitant deterioration in the fellow eye (6). Blood tests, including complete blood counts and thyroid function tests, were performed before treatment and one month after treatment, ensuring that the influence of glucocorticoid therapy on blood counts was minimized.

### 2.3 Clinical and laboratory assessments

Patient characteristics, including CAS, WBC, NEU, platelet (PLT), NLR, platelet-to-lymphocyte ratio (PLR), triglyceride (TG), total serum cholesterol (TC), and LDL, were collected from electronic medical reports. In addition, thyroid-stimulating hormone (TSH), free triiodothyronine (FT3), and free tetraiodothyronine (FT4) values should be collected from patients with GO. The severity of the condition was determined via the EUGOGO classification (mild, moderate-to-severe, and sight-threatening GO).

### 2.4 Statistical analysis

Owing to the distribution of the examined variables being close to normal and the large sample size (n > 100), parametric tests were used for statistical analysis. Continuous variables are presented as the means ± standard deviations (SDs) for each group, whereas categorical variables are presented as numbers and percentages (%). Student’s t test was used to compare differences between two groups, while one-way analysis of variance (ANOVA) was used for more than two groups, followed by the least significant difference (LSD) *post hoc* test. Because the CAS is an ordered categorical variable, the correlation analysis of the CAS was performed via Kendall’s Tau test. Univariate and multivariate logistic regression modeling was performed to identify variables that were significantly and independently associated with the occurrence of active GO and are presented as odds ratios (ORs) with 95% confidence intervals (CIs)).

The multivariate model was estimated via stepwise backward validation and contained only adjusted ORs for significant parameters. All p values are two-tailed, and p values < 0.05 were considered statistically significant. Receiver operating characteristic (ROC) curve analysis was subsequently performed. All calculations listed above were performed via SPSS 25.0.

Decision curve analysis (DCA) was conducted using R software packages with graphical capabilities. In DCA, the potential clinical benefit of a marker or model is evaluated by plotting net benefit against a range of threshold probabilities, thereby generating the decision curve. This method enables the identification of both the threshold probability range and the extent of clinical benefit for which the marker or model provides meaningful value.

## 3 Results

### 3.1 Baseline characteristics of the GO and control groups

**Table 1** summarizes the demographic and clinical data of the GO patients (n=220) and healthy controls (n=70). The mean ages of the patients in the active group (G1) were greater than those in the inactive group (G2) and the control group (G3). The mean age did not differ significantly between the G2 and G3 groups. The ratio of smokers in the active group was greater than that in the stable group (G1 *vs*. G2: 39/81 *vs*. 22/78, p<0.01). No significant differences were found in terms of body mass index (BMI) or sex distribution among the three groups. There were also no significant differences in the duration of GD (months) between the G1 and G2 groups.

**Table 1:** Baseline characteristics of the participants in the GO and control groups.

Parameters	G1	G2	G3	p-value	p-value	p-value
	n=120	n=100	n=70	(G1 *vs* G2)	(G2 *vs* G3)	(G1 *vs* G3)
Age(years)	48.42 ± 13.61	42.11 ± 12.49	43.38 ± 11.86	0.001	NS	0.013
Sex(Male/Female ratio)	62/58	51/49	35/35	NS	NS	NS
Smoking(Yes/No)	39/81	22/78	–	<0.001	–	–
BMI	25.9 ± 5.1	25.2 ± 4.9	25.5 ± 5.6	NS	NS	NS
Duration of GD (months)	61.32 ± 50.58	45.09 ± 59.85	–	NS	–	–

G1-Active GO group. G2-Inactive GO group. G3-Control group. BMI-body mass index. Continuous variables are presented as mean ± SD. Categorical variables are presented as number (percentage). p-values < 0.05 were considered as statistically significant. NS-no significance (p-values≥0.05. GO-Graves’ ophthalmopathy.

### 3.2 Differences in laboratory parameters among the active and inactive GO groups and the control group

**Table 2** shows the thyroid function indices of the patients in the G1 and G2 groups. The TRAb (13.49 ± 10.87 *vs*. 9.15 ± 10.49, p=0.01) and FT4 (22.43 ± 18.72 *vs*. 15.69 ± 12.52, p=0.008) values in the G1 group were significantly greater than those in the G2 group. There was no significant difference in FT3 or TSH between the G1 and G2 groups. Significant intergroup differences were observed in the mean values of WBC, NEU and NLR among the three groups, as shown in **Table 3**. Tukey’s *post hoc* analysis revealed significant differences in WBC (G1 *vs*. G2, p=0.038; G1 *vs*. G3, p<0.001; G2 *vs*. G3, p=0.005) and NEU (G1 *vs*. G2, p=0.014; G1 *vs*. G3, p<0.001; and G2 *vs*. G3, p=0.028) among the three groups. Additionally, statistically significant differences were observed in the PLT (231.07 ± 63.55 *vs*. 248.84 ± 59.70, p=0.84 ± 59.70, p=0.69 ± 1.71 *vs*. 2.31 ± 0.91, p=0.040) between G1 and G2, the NLR (2.69 ± 1.71 *vs*. 2.05 ± 0.64, p=0.001) between G1 and G3, and the PLT (248.84 ± 59.70 *vs*. 229.15 ± 49.41, p=0.041) between G2 and G3. There were no significant differences in the other indices among the three groups.

**Table 2:** Differences in the thyroid function indices between the active and inactive GO groups.

Parameters	G1	G2	P-value
TRAb (IU/l)	13.49 ± 10.87	9.15 ± 10.49	0.01
FT3 (ng/dL)	8.53 ± 6.47	7.06 ± 5.97	NS
FT4 (ng/dL)	22.43 ± 18.72	15.69 ± 12.52	0.008
TSH(mIU/L)	2.16 ± 6.89	3.34 ± 12.81	NS

G1-Active GO group. G2-Inactive GO group. P<0.05 was statistically significant. CAS-clinical activity score; TRAb-thyrotrophin receptor antibody; FT3-free triiodothyronine; FT4-free tetraiodothyronine; TSH-thyroid stimulating hormone; NS-no significance. (p-values ≥0.05).

**Table 3:** Differences in laboratory parameters between the GO groups and the control group.

Parameters	G1	G2	G3	P-value	P(G1 *vs*. G2)	P(G1 *vs*. G3)	P(G2 *vs*. G3)
WBC	6.54 ± 1.98	6.21 ± 1.34	5.40 ± 1.10	<0.001	0.038	<0.001	0.005
NEU	4.29 ± 1.81	3.80 ± 0.97	3.31 ± 0.90	<0.001	0.014	<0.001	0.028
LYM	1.80 ± 0.56	1.84 ± 0.57	1.67 ± 0.35	0.166	NS	NS	NS
PLT	231.07 ± 63.55	248.84 ± 59.70	229.15 ± 49.41	0.064	0.040	NS	0.041
PLR	141.44 ± 62.02	147.84 ± 60.05	142.80 ± 42.84	0.688	NS	NS	NS
NLR	2.69 ± 1.71	2.31 ± 0.91	2.05 ± 0.64	0.005	0.040	0.001	NS
NEUT	62.08 ± 13.25	61.64 ± 7.48	60.54 ± 6.57	0.593	NS	NS	NS
TG	1.50 ± 0.93	1.71 ± 1.27	1.36 ± 1.00	0.177	NS	NS	NS
TC	4.54 ± 1.23	4.67 ± 1.24	4.54 ± 0.61	0.879	NS	NS	NS
LDL	2.84 ± 0.93	2.89 ± 0.95	2.65 ± 0.59	0.202	NS	NS	NS

G1-Active GO group. G2-Inactive GO group. G3-Control group. P<0.05 was statistically significant. WBC-white blood cell; NEU-neutrophil; LYM-lymphocyte; PLT-platelet; PLR-platelet-to-lymphocyte ratio; NLR-neutrophil-to-lymphocyte ratio; NEUT-neutrophil ratio; TG-triglycerides; TC-serum total cholesterol; LDL-low-density lipoprotein; NS-no significance (p-values ≥0.05).

### 3.3 Univariate and multivariate regression analyses of the associations between laboratory parameters and the risk of active GO

To identify the parameters that were significantly relevant to active GO, logistic regression analysis was performed. The following four variables showed a significant relationship with GO in the univariate analysis: PLT (p = 0.033), NLR (p = 0.018), TRAb (p = 0.010), and FT4 p = 0.009). According to the univariate regression analysis, the following four variables were significantly related to active GO: PLT (p=0.019), NLR (p=0.006), TRAb (p=0.038), and FT4 (p=0.019) Notably, Smoking history was excluded as a confounding factor (p=0.070)(**Table 4**). The independent predictors (p < 0.05) in the above multivariate regression can be incorporated into the predictive model.

**Table 4:** Univariate and multivariate regression analyses of the associations between laboratory parameters and the risk of active GO.

Variable	Univariate		Multivariate	
	OR (95% CI)	p-Value	OR (95% CI)	p-Value
WBC	1.126(0.947-1.338)	0.180		
NEU	1.201(0.979-1.473)	0.079		
LYM	0.826(0.497-1.373)	0.461		
PLT	0.995(0.990-1.000)	0.033	0.993(0.987-0.999)	0.019
PLR	0.998(0.994-1.003)	0.489		
NLR	1.389(1.057-1.824)	0.018	1.689(1.158-2.464)	0.006
NEUT	1.002(0.976-1.029)	0.878		
TRAb	1.041(1.009-1.073)	0.010	1.034(1.002-1.068)	0.038
TG	0.879(0.669-1.155)	0.356		
TC	0.911(0.709-1.171)	0.469		
LDL	0.936(0.673-1.302)	0.693		
FT3	1.043(0.990-1.099)	0.111		
FT4	1.032(1.008-1.057)	0.009	1.033(1.005-1.061)	0.019
TSH	0.988(0.955-1.021)	0.465		
Smoking	0.562(0.300-1.051)	0.070		

P<0.05 was statistically significant. OR-odds ratio; CI-confidence Interval; WBC-white blood cell; NEU-neutrophil; LYM-lymphocyte; PLT-platelet; PLR-platelet-to-lymphocyte ratio; NLR-neutrophil-to-lymphocyte ratio; NEUT-neutrophil ratio; TG-triglycerides; TC-serum total cholesterol; LDL-low-density lipoprotein.

### 3.4 Correlations between laboratory parameters and CAS scores

In the GO group, Kendall’s tau test revealed significant correlations between CAS scores and laboratory parameters (WBC, NEU, NLR, TRAb, FT3, FT4 and TSH), the results of which are presented in **Table 4**. The strength of the monotonic positive relationship reflected by the Kendall correlation coefficient was r= 0.155 for WBC, r = 0.165 for NEU, r = 0.130 for NEUT, r = 0.134 for NLR, r = 0.160 for TRAb, r = 0.117 for FT3, and r = 0.139 for FT4, and the monotonic negative relationship was r = -0.114 for TSH (**Table 5**).

**Table 5:** Correlations between laboratory parameters and CAS scores in the GO groups.

Parameter	r-value (p value)
WBC	0.155 (0.003)
NEU	0.165 (0.002)
LYM	-0.021 (0.695)
PLT	-0.059 (0.259)
NEUT	0.130 (0.013)
NLR	0.134 (0.010)
PLR	-0.027 (0.607)
TG	-0.005 (0.923)
TC	-0.026 (0.646)
LDL	-0.006 (0.921)
TRAb	0.160 (0.004)
FT3	0.117 (0.031)
FT4	0.139 (0.011)
TSH	-0.114 (0.043)

P<0.05 was statistically significant. WBC-white blood cell; NEU-neutrophil; LYM-lymphocyte; PLT-platelet; PLR-platelet-to-lymphocyte ratio; NLR-neutrophil-to-lymphocyte ratio; NEUT-neutrophil ratio; TG-triglycerides; TC-serum total cholesterol; LDL-low-density lipoprotein; TRAb-thyrotrophin receptor antibody; FT3-free triiodothyronine; FT4-free tetraiodothyronine; TSH-thyroid stimulating hormone.

### 3.5 ROC analysis, multifactor prediction model and DCA curve

**Figure 1:**
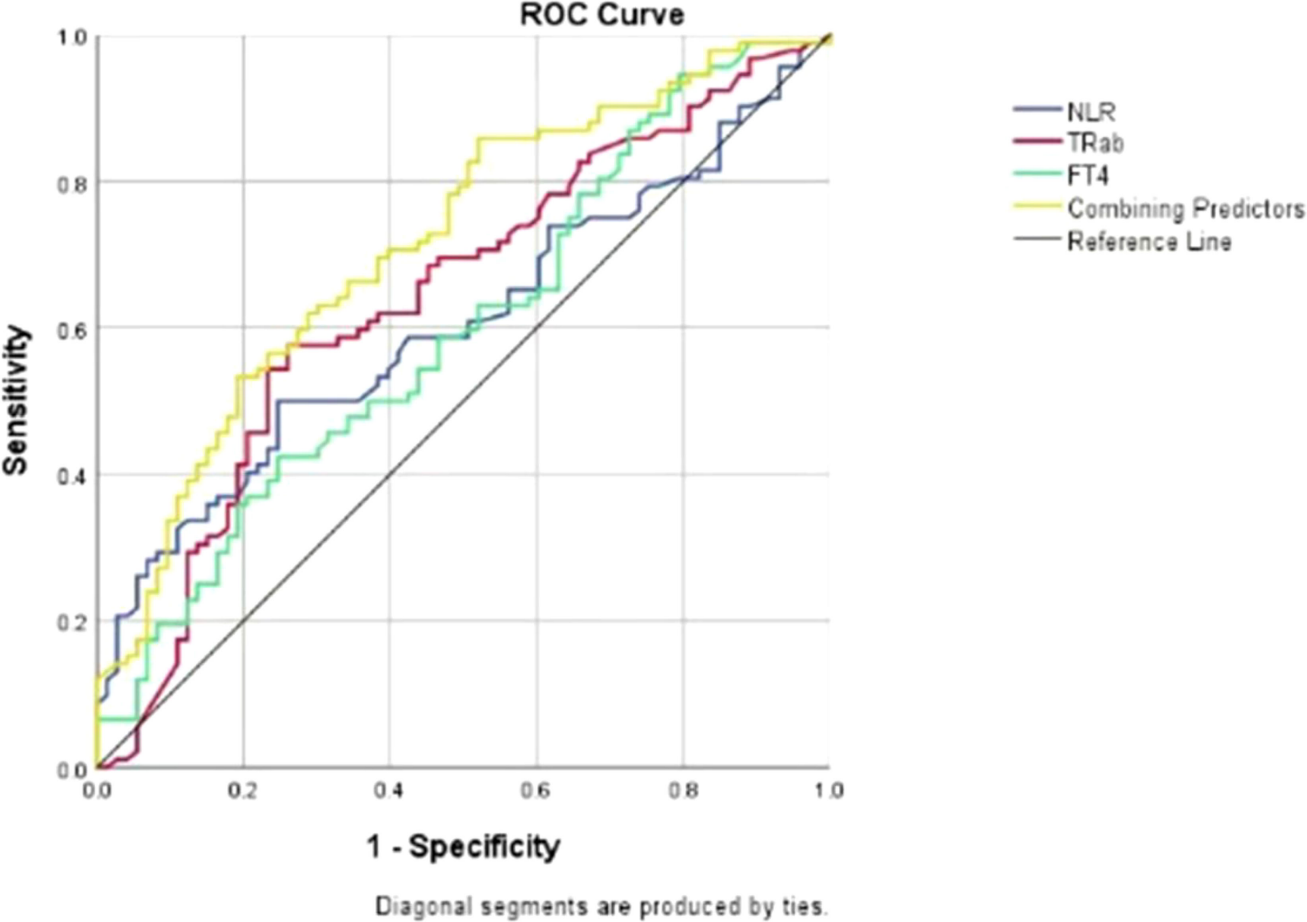
Receiver operating characteristic (ROC) curves for TRAb, FT4, NLR and their combination as predictors of disease activity in patients with GO. *ROC- receiver operating characteristic; NLR-neutrophil-to-lymphocyte ratio; TRAb-thyrotrophin receptor antibody; FT4-free tetraiodothyronine*.

**Figure 2:**
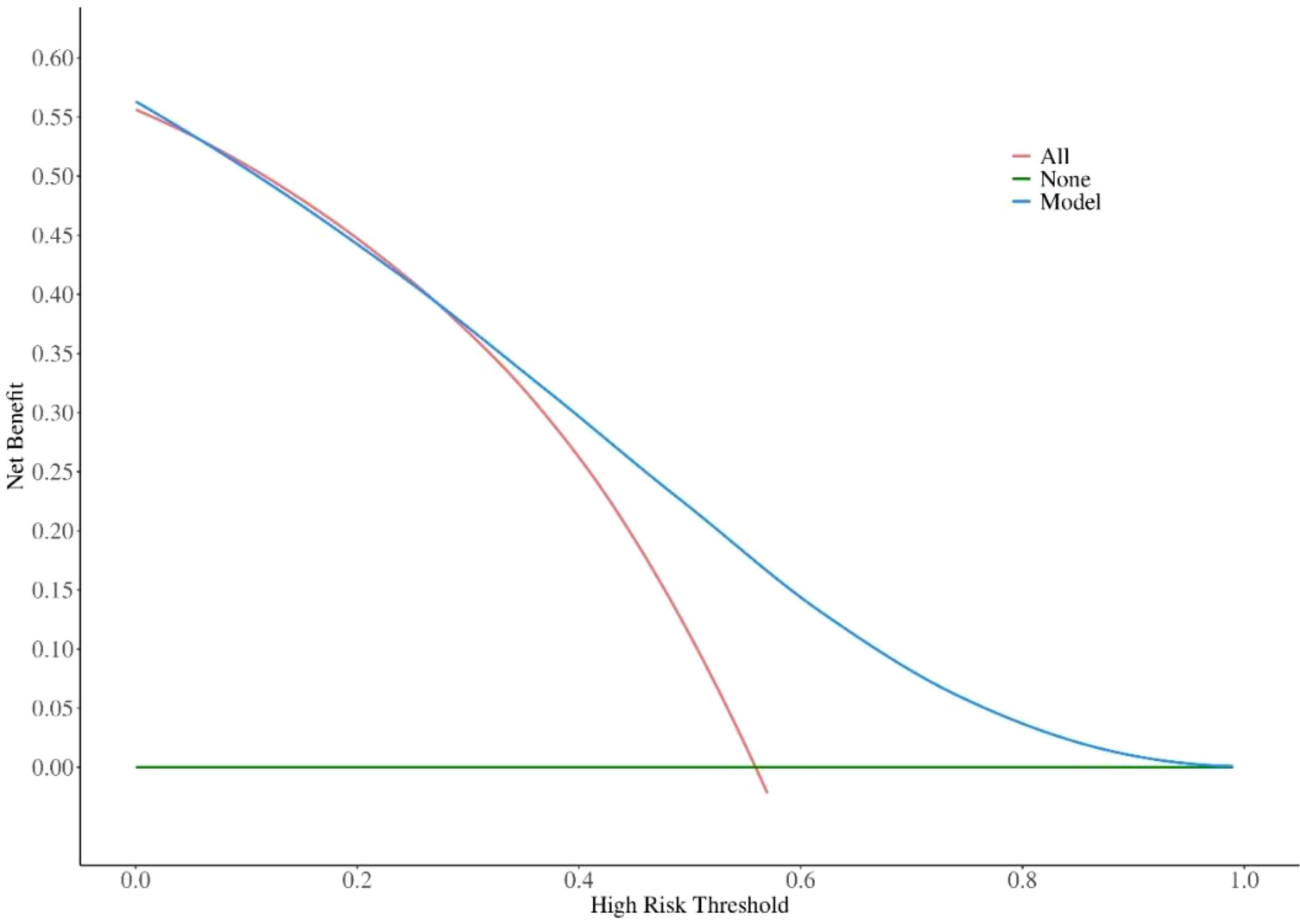
Decision Curve Analysis (DCA) of the Prediction Model. DCA, Decision Curve Analysis.

The overall area under the receiver operating characteristic curve analysis of the biomarkers predicting the activity of GO is shown in **Figure 1**. For TRAb, FT4, NLR and their combined predictors, ROC analysis revealed area under the curve (AUC) values of 0.643, 0.606, 0.597 and 0.716, respectively, for discriminating active from inactive phases. Using Youden’s index, the optimal cutoff values were determined as follows: 8.71 IU/l for TRAb, with a sensitivity of 0.58 and specificity of 0.74; 19.82 ng/dL for FT4, with a sensitivity of 0.41 and specificity of 0.75; 2.405 for the NLR, with a sensitivity of 0.50 and specificity of 0.75; and 0.6 for combining predictors, with a sensitivity of 0.533 and specificity of 0.808. These results demonstrate that the combination model has better sensitivity and specificity in predicting the activity of GO compared with single factors. In addition, the DCA shows that the net gain reaches its maximum at 15-40% within the threshold probability range of 30%-60% (**Figure 2**).

### 3.6 Validation of the prediction model

For the 15 patients with active GO who responded well to IVMP treatment, the NLRs (before *vs*. after: 2.94 ± 1.03 *vs*. 2.15 ± 0.65, P=0.045), TRAb levels (before *vs*. after: 18.20 ± 10.50 *vs*. 7.40 ± 6.34, P=0.002), and FT4 levels (before *vs*. after: 27.80 ± 22.96 *vs*. 12.80 ± 6.11, P=0.004) significantly increased before IVMP treatment but decreased when the patients reached the inactive stage after treatment (all P<0.05).

## 4 Discussion

This study comprehensively evaluated the differences in the expression of peripheral blood markers between active and inactive GO patients and revealed that several laboratory markers, such as TRAb, FT4, and the NLR, were closely related to GO activity. Given that these markers may be useful to predict disease activity (14), we combined the above three markers to establish a prediction model, which demonstrates predictive potential for GO progression under observational conditions.

Peripheral blood markers are increasingly used as prognostic tools in autoimmune diseases and cancers owing to their clinical utility (13, 15–17), though their role in Graves’ orbitopathy remains unclear. Szydełko J et al. reported significant increases in the WBC and NEU counts and the NLR in GO patients compared with those in GD-only patients or healthy individuals, which aligns with our findings (11). Similar results were also obtained by Celik et al. (12), who reported the involvement of nearly all subpopulations of WBCs in the pathogenesis of GO. However, the mechanisms behind changes in blood cell counts remain unclear. T-cell activation is crucial in autoimmunity development. These findings suggest that T lymphocytes stimulate B lymphocytes to produce autoreactive antibodies, triggering fibroblast proliferation in the orbit and causing GO symptoms. Significantly, activated T cells play a key role in GO pathogenesis by releasing cytokines like, such as tumor necrosis factor-alpha (TNF-α), interleukin-1 beta (IL-1β), interleukin-6 (IL-6), and interleukin-17 (IL-17) (18). These cytokines drive the production and recruitment of neutrophils and macrophages, helping explain the increases in WBC, NEU, LYM counts, and NLR during GO progression (18).

Our study confirms previous findings and explores their implications for CAS. Elevated WBC, NEU, and NLR are correlated with increased disease activity. Multivariate regression and ROC analyses show that an elevated NLR reliably predicts GO progression to its active phase. If NLR exceeds 2.405, clinical evaluation should be prioritized to prevent rapid progression. Generally, the presence of neutrophilia coupled with lymphocytopenia is indicative of tissue damage, stress, and inflammation (19). Thus, a high NLR signifies an unfavorable prognosis across various inflammatory conditions (20–22). Several studies suggest that NLR may serve as an inflammatory marker in thyroid and other diseases (23–26). Keskin et al. reported that the NLR was significantly greater in euthyroid chronic autoimmune thyroiditis patients than in healthy controls and was positively correlated with disease-related autoantibodies (27). These findings corroborate the utility of the NLR as a predictive indicator of GO activity.

GO is recognized as an autoimmune thyroid disorder (AITD) caused by unregulated TRAb synthesis. Our study confirmed that TRAb levels were significantly elevated in GO patients and strongly correlated with CAS (28–30). Furthermore, logistic multivariate analysis indicated that TRAb was a strong predictor of active GO and our model identifies levels >8.71 IU/L serving as a key threshold for predicting progression. In accordance with our findings, Diana and Nicoli reported a close relationship between TRAb concentrations and the clinical activity and severity of GO, underscoring a statistically significant direct correlation between serum TRAb levels and CAS (29). Zhao et al. (31) investigated the role of TRAb in the diagnosis and prediction of GO, revealing a positive correlation between TRAb levels and GO occurrence. In the ROC curve analysis for GO prediction, TRAb demonstrated an AUC of 0.719, which was slightly higher than our result (AUC=0.643). Our findings corroborate this observation. Given the documented overexpression of TSHR in orbital tissues of patients with GO (30), TRAb levels are considered independent risk factors and biomarkers for GO (32):

In addition to the factors mentioned above, unstable thyroid function has emerged as a significant risk factor for GO (1). Currently, there are few prediction models for GO that incorporate FT4 levels. Wang et al. (33) developed a predictive model for refractory GD and identified that elevated FT4 levels were significantly associated with an increased risk of refractory disease. In our investigation, a majority of patients in both the inactive and active phases were diagnosed with unstable hyperthyroidism. Furthermore, we observed a positive correlation between FT4 levels and CAS, which is consistent with the clinical consensus that inadequate control of thyroid function can precipitate the progression of GO. This assertion was substantiated by our multiple regression analysis, indicating that elevated FT4 levels may indeed serve as a risk factor for the progression of GO. More importantly, our study establishes FT4 levels exceeding 19.82 ng/dL may exhibit a higher likelihood of progressing to active GO.

Interestingly, our study revealed that the PLT in inactive GO patients was greater than that in active GO patients and healthy subjects, but no correlation was detected between the PLT count and CAS. Some researchers believe that GD is an autoimmune disease that may cause thrombocytopenia during the course of the disease through immune-mediated platelet destruction or splenic retention (28). However, we had difficulty interpreting the observation that the PLT in the control group was lower than that in the inactive phase of GO. This discrepancy may be due to selection bias and an insufficient sample size. Notably, Taskaldiran et al. (34) did not find any difference in the PLT between the GO patients and the control group. Therefore, given the conflicting results of multiple studies, whether PLT can predict GO activity requires further investigation.

Based on the results, we developed a prediction model combining three biomarkers and validated it. When the NLR, TRAb, and FT4 are elevated above 2.405, 8.71 IU/l and 19.82 ng/dL, respectively, the likelihood of the GO being in the active phase is 60%. The combined predictor area under the ROC curve was greater than any individual factor, indicating better prediction of GO progression. Despite lower sensitivity (0.533), the moderate AUC (0.716) and balanced specificity (0.808) suggest the model is useful in minimizing false positives, which can lead to overtreatment and burden patients. Additionally, DCA confirmed the model’s clinical utility, providing the highest net benefit (15%-40%) at decision threshold probabilities of 30%-60% (typical for intermediate-risk patients). We also retrospectively analyzed 15 patients who responded well to IVMP from January to August 2024. NLR, TRAb, and FT4 were elevated in active GO patients before treatment and decreased when they reached the inactive phase after treatment (P<0.05). While the relationship between these biomarkers and IVMP response remains unclear, further research is needed to use these indicators to predict treatment response in GO patients.

Other hematological indicators also have some predictive value for GO. Previous studies on blood lipids have focused mainly on differences between patients with GD and controls. TC and LDL levels have been shown to be independent risk factors for GO (32, 35–38). Current guidelines recommend cholesterol-lowering therapy for all patients with Graves’ disease (32) to prevent the development of GO. We noted that the average levels of TC, LDL and TG were greater in GO patients than in controls, although these differences were not statistically significant. This trend may be attributed to the greater prevalence of hyperthyroidism (elevated concentrations of FT3 and FT4) in this patient cohort. However, to substantiate this hypothesis, further investigations with larger sample sizes are imperative.

In addition to common blood biomarkers mentioned above, emerging biomarkers like circulating miR-146a also contribute to predicting active GO. Recent studies have demonstrated that circulating may predict glucocorticoid treatment response in GO patients (35). As a crucial “inflammatory regulatory RNA”, miR-146a modulates immune responses via pathways like NF-κB and NLRP3 inflammasome (39, 40). A recent study reported that pre-treatment serum levels of miR-146a were significantly associated with the treatment outcomes in GO patients, suggesting miR-146a is a simple, objective, and reliable biomarker that may provide new references for clinical decision-making (35).However, limitations such as high detection costs and small sample sizes (n<100) exist. Future multi-center cohort studies (planned n=500) are needed to systematically evaluate their clinical utility and explore cost-effective detection methods.

Moreover, numerous studies have consistently reported a greater incidence of GO in females than in males (27, 28). However, our study revealed similar sex ratios in GO patients. This may be because severe GO patients predominated among patients seeking hospitalization and treatment, while the sex difference in severe thyroid-related eye disease was smaller (29). Therefore, we believe that sex may not be a significant risk factor for the progression of GO.

Finally, we observed that the average age of patients in the active stage was significantly greater than that of patients in the inactive stage. However, no studies have confirmed the exact association between age and GO activity. Some studies have shown that the duration of thyroid dysfunction may affect the occurrence and progression of GO (30).Due to the retrospective nature of this study, recall bias affected disease reporting, and patients could not accurately identify the timeline from GD to GO. As a result, we excluded GO duration from the regression analysis. Future prospective studies should include longitudinal follow-up of GO duration as a potential predictor. In addition, the ratio of smokers in the active GO group was greater than that in the inactive GO group in our study. This confirmed the findings of previous studies that smoking is a risk factor for active GO (30). While our study eliminated smoking as a confounder in univariate analysis (possibly owing to recall bias and exposure quantification challenges), its inclusion remains warranted in clinical risk assessment.

Overall, our model demonstrates greater reliability compared to the aforementioned single blood biomarkers, while remaining cost-effective and easily accessible. Furthermore, it maintains multiple advantages over traditional assessment models: such as the EUGOGO criteria, depend on variables like smoking status, TSH levels, and CAS scores but lack molecular precision. In contrast, our NLR/TRAb/FT4 biomarker-based model demonstrates superior early risk stratification [AUC 0.72 *vs*. 0.67 (41) in retrospective cohorts]. Meanwhile, emerging imaging-based AI models, including orbital MRI radiomics (e.g., T2-weighted texture analysis), achieve competitive performance [AUC 0.86 (42)] but are limited by specialized equipment requirements, whereas our serum-based approach offers greater accessibility.

There are several limitations of this study. First, this was a retrospective, single-center study. Therefore, a large prospective validation study is needed. Second, this study only used the CAS to differentiate GO patients. Although the CAS is the most commonly used and best validated scoring system for disease activity, it also has some limitations (35). Some scholars have suggested the use of other methods, such as magnetic resonance imaging (MRI) (6), could grade disease activity. Owing to the high cost of this examination, the included cases did not fully cover this examination, and our study did not include this examination in the analysis. Future prospective cohort studies that combine orbital MRI with CAS scores to assess GO activity more objectively are needed. Third, our validation procedures were conducted within our study; further external validation is needed to evaluate the generalizability of our prediction model.

In conclusion, we developed a model to predict GO activity and progression by combining three factors, NLR, FT4, and TRAb, based on univariate and multivariate regression analyses. This prediction model appears to improve the predictive performance of single-activity biomarkers and may help to assess GO activity and progression in a timely manner.

## Data Availability

The raw data supporting the conclusions of this article will be made available by the authors, without undue reservation.
